# Quasispecies of SARS-CoV-2 revealed by single nucleotide polymorphisms (SNPs) analysis

**DOI:** 10.1080/21505594.2021.1911477

**Published:** 2021-05-25

**Authors:** Rongsui Gao, Wenhong Zu, Yang Liu, Junhua Li, Zeyao Li, Yanling Wen, Haiyan Wang, Jing Yuan, Lin Cheng, Shengyuan Zhang, Yu Zhang, Shuye Zhang, Weilong Liu, Xun Lan, Lei Liu, Feng Li, Zheng Zhang

**Affiliations:** a Institute for Hepatology, National Clinical Research Center for Infectious Disease, Shenzhen Third People’s Hospital, the Second Affiliated Hospital, School of Medicine, Southern University of Science and Technology, Shenzhen, Guangdong Province, China; b Shenzhen Key Laboratory of Unknown Pathogen Identification, BGI-Shenzhen, Shenzhen, China; c School of Life Sciences, Tsinghua University, Beijing, China; d Department of Infectious Diseases, Shenzhen Third People’s Hospital, National Clinical Research Center for Infectious Disease, Shenzhen, Guangdong Province, China; e National Supercomputing Center in Shenzhen (Shenzhen Cloud Computing Center), Shenzhen, China; f Shanghai Public Health Clinical Center, Institute of Biomedical Sciences, Fudan University, Shanghai, China; g Department of Basic Medical Sciences at School of Medicine, Tsinghua University, Tsinghua-Peking Center for Life Sciences, Tsinghua University, Beijing, China; h Guangzhou Eighth People’s Hospital, Guangzhou Medical University, Guangzhou, Guangdong, China; i Shenzhen Research Center for Communicable Disease Diagnosis and Treatment of Chinese Academy of Medical Science, Shenzhen, Guangdong Province, China; j Guangdong Key Laboratory for Anti-infection Drug Quality Evaluation, Shenzhen, Guangdong Province, China

**Keywords:** Single nucleotide polymorphism (SNP), intra-host single nucleotide variation (iSNV), SARS-CoV-2, quasispecies, selective pressure, host adaptation, epitopes

## Abstract

New SARS-CoV-2 mutants have been continuously indentified with enhanced transmission ever since its outbreak in early 2020. As an RNA virus, SARS-CoV-2 has a high mutation rate due to the low fidelity of RNA polymerase. To study the single nucleotide polymorphisms (SNPs) dynamics of SARS-CoV-2, 158 SNPs with high confidence were identified by deep meta-transcriptomic sequencing, and the most common SNP type was C > T. Analyses of intra-host population diversity revealed that intra-host quasispecies’ composition varies with time during the early onset of symptoms, which implicates viral evolution during infection. Network analysis of co-occurring SNPs revealed the most abundant non-synonymous SNP 22,638 in the S glycoprotein RBD region and 28,144 in the ORF8 region. Furthermore, SARS-CoV-2 variations differ in an individual’s respiratory tissue (nose, throat, BALF, or sputum), suggesting independent compartmentalization of SARS-CoV-2 populations in patients. The positive selection analysis of the SARS-CoV-2 genome uncovered the positive selected amino acid G251V on ORF3a. Alternative allele frequency spectrum (AAFS) of all variants revealed that ORF8 could bear alternate alleles with high frequency. Overall, the results show the quasispecies’ profile of SARS-CoV-2 in the respiratory tract in the first two months after the outbreak.

## Introduction

RNA viruses have exhibited high mutation rates primarily due to the low-fidelity of RNA polymerases [1] and the absence of post-replication nucleotide repair mechanisms [2,3]. Therefore, RNA viruses always exist as populations of viral variants containing different mutations between the hosts (inter-host) or within an individual host (intra-host), referred to as quasispecies [4,5]. Quasispecies is believed to be a strategy of virus evolution [3] and has previously been reported in the SARS-CoV [6] and MERS-CoV [7] viruses.

The phylogenetic analysis of the SARS-CoV-2 strains spread in different regions of the world confirmed its frequent recombination with other human coronaviruses or coronaviruses from pangolins and bats [8,9]. According to amino acid sequence mutations, an early survey classified the various virus strains across the world into three clusters: cluster A, cluster B (T8782C/C28,144T), and cluster C (G26,144T/G251V, ORF3a)[10]. The mutation rate of SARS-CoV-2 has been estimated as ~6 × 10^−4^ (CI: 4 × 10^−4^ ~ 7 × 10^−4^) nucleotides/genome/year[11]. So far, typically acquired SARS-CoV-2 only goes through one to two mutations per month[12], which is largely unremarkable for an RNA virus [11,13,14]. SARS-CoV-2 strains have also been widely distributed in the global phylogeny with high diversity. According to GISAID nomenclature, the mutations in SARS-CoV-2 genomes divide into different clades, such as S clade (L84S in ORF8, U28,144C in the genome), V clade (G251V in ORF3a, G26,144U), and G clade (characterized by D614G in the S protein, A23,403G)[15]. Recently, a dynamic nomenclature proposal for SARS-CoV-2 lineages has been suggested. The sequences that share nucleotides at position 8782 (U) and 28,144 (C) with the closest known bat virus RaTG13 are defined as lineage A, while the sequences with 8782 (C) and 28,144 (T) were defined as lineage B, namely the lineage A/B nomenclature[16]. A new variant, however, was just discovered in the United Kingdom, named B.1.1.7, which is a fast-spreading variant with increased interaction force between Spike-ACE2 caused by the viral N501Y mutation [17,18]. These new variants are alarming, suggesting that the virus is rapidly evolving and adapting to transmit rapidly in the population.

Although the evolutionary history and transmission dynamics of SARS-CoV-2 have been gradually clarified, more work is still needed to explore the inter-host and intra-host variations of SARS-CoV-2, as such variations may point to the direction of the evolution of the viral genome for the adaptation to the host’s immune response[19], and can help in the development of antiviral drugs[20], and other selective pressures, for instance, the widespread use of vaccines, X-ray radiation therapy[21], and public health intervention strategies. Moreover, such variations contribute significantly to the design of effective strategies for disease control and prevention.

The immune system plays a vital role in the defense against SARS-CoV-2 infection[22], since none of the drugs used to treat coronavirus disease 2019 (COVID-19) can directly clear the virus *invivo*[23]. Therefore, the host’s immune system is also an important factor in determining the virus’s evolution. The humoral response and cellular response work together to inhibit the virus’s replication and prevent damage caused by excessive immunity to the host [24–26]. Virus-specific serum antibodies (Abs) are important adaptive humoral immune responses against viral infection[23]. Protection-specific Abs, including immunoglobulin G (IgG) Abs and neutralizing Abs (NAbs), are produced by B cells after infection with the virus, blocking the virus from entering the host cells and defending against viral reinfection[27]. The structural proteins of SARS-CoV-2 are potential epitopes of Abs, especially the S protein. The S-specific NAbs have been detected in recovered COVID-19 patients’ serum [28–31]. T-cell responses are also essential for adaptive immunity against viral infections *in* *vivo*. CD8^+^ CTL can kill virus-infected host cells by recognizing MHC-presented viral peptides from virus-infected cells. CTL epitopes have been identified in SARS-CoV-2 surface glycoprotein[32]. Thus, it is rational to hypothesize that as the virus interacts with the host’s immune system, the virus may evolve and viral mutation sites may be the epitopes of the host’s immune system.

During infection, further exploration may be needed to identify whether a viral quasispecies infects the host or the virus evolves in-vivo to form quasispecies after invading the host. It is well known that genetic diversity in pathogen’s quasispecies is influenced by pathogen-host interaction to adapt to different hosts and tissues, which have been observed both in viruses and bacteria. For instance, in *Helicobacter pylori* in mouse samples, multiple single nucleotide polymorphisms were found in its virulence factor region through intra- and inter-genomic variation analysis[33]. The quasispecies composition of Influenza A in the membrane glycoproteins hemagglutinin (HA) region changed during adaptation processes from Vero cells to Madin-Darby Canine Kidney (MDCK) cells[34]. The dynamics of rabies virus quasispecies during serial passage have also been identified in heterologous hosts[35]. As for SARS-CoV-2, it can cause infection in both the upper respiratory tract (URT) and the lower respiratory tract (LRT) and the viral kinetics of SARS-CoV-2 infection are related to infectiousness and disease progression[36]. More so, the URT is specialized in eliminating inhaled pathogens to prevent viral invasion in the lower respiratory tract[37]. Research has also aimed to identify the different protective mechanisms between the upper and lower respiratory tracts[38]. In the URT, this is mainly mediated by specific IgA- and IgG2a-producing B cells. In contrast, ex-vivo active effector memory CTL was found in the LRT[38]. Thus, it is necessary to study the genetic diversity of SARS-CoV-2 in the upper and lower respiratory tracts.

This study aimed to reveal the genetic diversity in SARS-CoV-2 quasispecies in human specimens and obtain new insights into the impact of distinct environments on virus evolution. To this end, we took advantage of meta-transcriptomic sequencing to perform a comparative analysis of genomic diversity of SARS-CoV-2, sampled at different time points during symptom onset in the upper (nasal swabs, throat swabs, and sputum) and lower (Bronchoalveolar Fluid (BALF)) respiratory tract of patients. These analyses assessed the microevolution profile of SARS-CoV-2 during infection and indicated mutations useful for viral adaptation.

## Materials and methods

### Ethics and patient sampling

This study was conducted according to the principles expressed in the Helsinki Declaration. Ethical approval was obtained from the Research Ethics Committee of the Shenzhen Third People’s Hospital (2020–192). Written consent was obtained from patients or their guardian when samples were collected. Patients were informed before providing written consent and data directly related to disease control were collected and de-identified for analysis.

### Sampling, RNA extraction, and RT-qPCR

All 48 COVID-19 patients in this study were enrolled from the Shenzhen Third People’s Hospital from January 20–30, 2020. The severity of the disease was classified as mild or severe based on the Diagnosis and Treatment Scheme of SARS-CoV-2, released by the National Health Commission of China. Throat swabs, sputum, nasal swabs, and supernatant of bronchoalveolar lavage fluid (BALF) were collected from patients at various time points and were sent to the diagnostic laboratory. Total nucleic acid was extracted from different samples using QIAamp Viral RNA Mini Kit (Qiagen, Cat. No. 52,904). Real-time reverse transcription PCR (qRT-PCR) targeting ORF-1a/b and N of SARS-CoV-2 was performed using a China Food and Drug Administration (CFDA) approved commercial kit following the manufacture’s protocol (GeneoDX Co., Ltd., Shanghai, China).

### Meta-transcriptome libraries preparation and sequencing

The positive nucleic acid extractions were treated by DNase I (NEB, Cat. No. M0303S) to remove the host DNA. The concentrations of all isolated RNA samples were measured with Qubit RNA HS Assay Kit (Thermo Fisher Scientific, Waltham, MA, USA). Libraries were prepared using the MGIEasy RNA Library preparation Kit v2 (MGI, Cat. No. 1000005,953) as follows: (1) RNA was fragmented by incubating with fragmentation buffer at 87 °C for 6 minutes; (2) ds cDNA was synthesized using random hexamers with fragmented RNA; (3) ds cDNA was subjected to end repair, adaptor ligation, and 18-cycle PCR amplification; and (4) PCR products were unique dual indexed (UDI) before going through circularization and rolling circle replication (RCR) to generate DNA nanoball (DNB)-based libraries. Negative controls were prepared from nuclease-free water. DNB preps of clinical samples were sequenced on the ultra-high-throughput DNBSEQ-T7 platform (MGI, Shenzhen, China) with a paired-end 100 nt strategy, generating, on average, 321 Gb sequencing data for each sample.

### SNP calling analysis

Raw reads were trimmed by trim galore v 0.6.4 [39] with default parameters to remove low-quality bases with a score of less than 25. Trimmed reads were mapped to the reference SARS-CoV-2 genome (Accession: MN908947.3) using BWA-MEM[40]. About 99.9% of reads that could not be aligned were removed. Duplicates in the bam files were removed by Samtools v1.10[41]. Variants were called by HaplotypeCaller in the Genome Analysis Toolkit (GATK, v4.1.4.1)[42], the vcf files for each separate sample were combined by GATK CombineGVCFs, and then genotyped by GATK GenotypeGVCFs. Single nucleotide variants (SNVs) were further filtered by GATK SelectVariants with the parameter of QD < 2.0, FS > 200.0, SOR > 10.0, MQRankSum < −12.5, and ReadPosRankSum < −8.0. The VCF output generated by GATK were then parsed by a home-made R script. The allele frequencies of SNP were calculated using only those with the sequencing depth > 10x and altered base > 2x. SNP is filtered based on the following criteria: minor allele is sequenced at least 2 times and has a minimum frequency (alternate frequency) of 1%.

### Positive selection analysis of SARS-CoV-2 genes

The nucleotide sequences of S, M, N, ORF3, ORF8, and ORF10 genes, which were obtained from sample sequencing were aligned using Mega5.0[43]. Duplicate gene sequences were removed. Phylogenetic analysis of each gene was performed using an approximate maximum likelihood method implemented in FastTree 2.1 [44] with the WAG+CAT model. Likelihood ratio test of positive selection was performed by comparing M7 (beta) and M8 (beta & ω) models using PAML4.9 software package [45,46]. Sites under positive selection were identified using the Bayes empirical Bayes (BEB) procedure. For evaluation of dN/dS ratio at the individual gene level, the SNP data in our dataset was used to construct the alternative sequence for each gene of SARS-CoV-2 using MN908947.3 as the reference. The homologous sequences were aligned using MUSCLE. The dN and dS were calculated using the Nei-Gojobori method [47], with the JC substitution model implemented in DnaSP 5[48].

### The Alternative Allele Frequency Spectrum (AAFS) of SARS-CoV-2 genes

We selected the SNPs annotated as non-synonymous mutation in the SNP calling analysis. Then we grouped these SNPs according to their genomic locations on the different genes. The alternative allele frequencies of the SNPs were obtained from the SNP calling analysis and the cumulative distribution of the alternative allele frequencies of all SNPs for each gene through the stat_ecdf function in R package ggplot2 was calculated [49] Additionally, the mean value of the alternative allele frequencies for each SNP shared in different samples was calculated in order to represent the frequency of each unique SNP and to avoid the dominant influence that the shared SNPs in some samples may have on the cumulative curve. Lastly, the cumulative distribution for these SNPs based on the average frequency among samples was calculated.

### Network analysis

**Figure 1.**
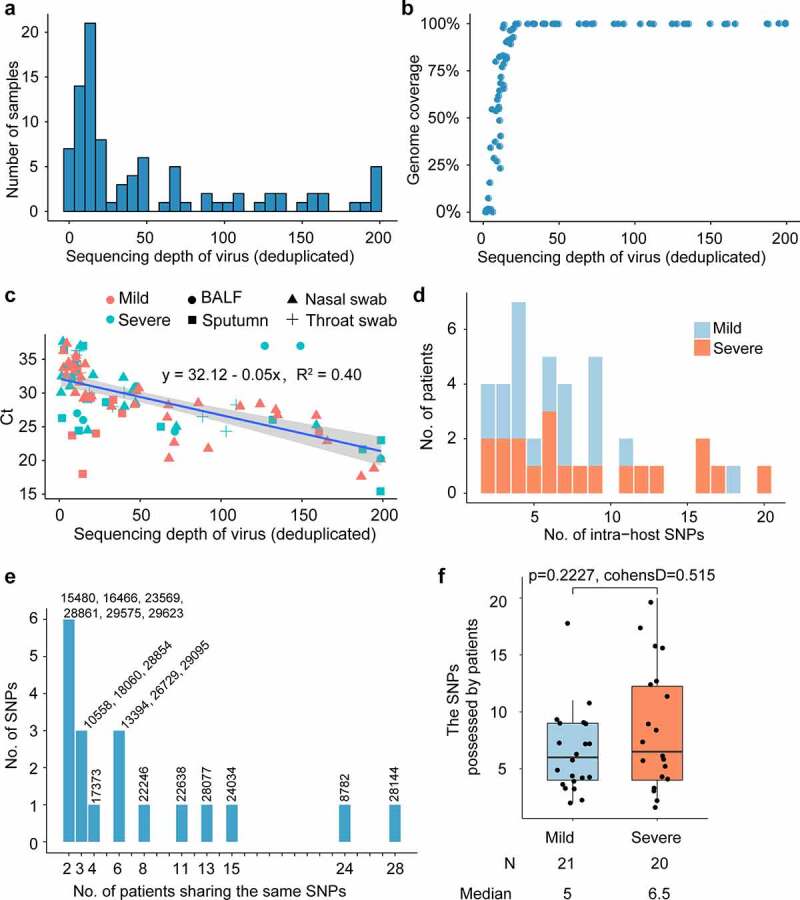
Distribution of SNPs among COVID-19 patients

Networks of the samples sharing SNPs were constructed using Cytoscape[50], with each node representing a sample. Two nodes were connected by an edge if they shared at least one SNP. The layout was determined using an edge-weighted spring-embedded model. There are 63 nodes and 1047 edges in the network. Among the 70 samples in which SNPs were identified, 7 samples did not share any SNPs. The samples collected from the same patient were treated as independent samples, as they were collected from different tissues of the respiratory tract. Among these commonly shared SNPs, SNPs at 22,638 were identified in 16 samples collected from 11 patients; SNPs at 28,144 were identified in 42 samples collected from 28 patients; and SNPs at 8782 were identified in 36 samples collected from 24 patients. The number of patients sharing the same SNPs is shown in (Figure 1e).

A. Sequencing depth of SARS-CoV-2 for 94 samples. **B**. The plot of SARS-CoV-2 sequencing depth against genome coverage for the 94 samples. The y-axis represents genome coverage which is calculated as the propotion of length covered by mapped reads accounting for the whole length of the reference genome MN908947.3. **C**. The plot of SARS-CoV-2 sequencing depth against RT-PCR Ct value for the 94 samples from different organs of patients with different severity. The y-axis represents CT values of samples which reflect viral load (The CT value is inversely proportional to the viral load). The x-axis represents sequencing depth of every sample which is calculated as bases of all mapped reads divided by bases of reference genome MN908947.3. The sequencing depth of the virus in the sample is proportional to the viral load. **D**. Severe and mild patients have SNPs. **E**. The number of patients share the same SNPs. **F**. Box plot of the SNPs in mild patients and severe patients. The number of SNPs possessed by mild and severe patients is compared to each other with the Student’s t-test (t.test in R 3.6.2, two-sided, unadjusted for multiple comparisons).

### Coimmunoprecipitation (Co-IP) to detect the interaction of ORF8 and HLA-A2

The pierce classic IP kit (Thermo scientific 26,146) was used to perform Co-IP assay. In brief, HEK293T cells were lysed with IP lysis buffer (0.025 M Tris, 0.15 M NaCl, 0.001 M EDTA, 1% NP-40, 5% glycerol, 1% protease inhibitor cocktail, pH 7.4) (TransGen Biotech) for 15 minutes on ice with brief vertaxing every 5 minutes. Then pre-clear lysates were prepared using the control agarose resin. The pre-clear lysates were collected and incubated with 2–10 μg anti-FLAG antibody overnight at 4 °C with rotating to form immune complexes. Protein A/G plus agarose was added to the antibody/lysate sample and the incubated mixture was gentle shook for an hour. The immunoprecipitates were washed 4 times with ice-cold STN buffer, eluted by boiling SDS loading buffer and separated by SDS-PAGE for western blotting.

### Western blotting for detection of ORF8 and HLA-A2

Cell lysates were prepared 24 hours after co-transfect (Bio-Rad). The samples were boiled for 5 minutes, separated by SDS-PAGE, and transferred to PVDF membranes. The membranes were blocked with 5% nonfat dry milk in Tris-buffered saline supplemented with 0.5% Tween 20, and proteins were detected by incubation with primary antibodies diluted in blocking buffer, followed by incubation with secondary antibodies (raised in goat against the appropriate species) conjugated to horseradish peroxidase (HRP) and diluted in blocking buffer. GST was detected using a rabbit polyclonal anti-GST antibody (catalog no. ab19256; Abcam), FLAG was detected using mouse polyclonal anti-FLAG antibodies (catalog no. HT201-01; TransGen Biotech), and GAPDH (glyceraldehyde-3-phosphate dehydrogenase) was detected using mouse monoclonal anti-GAPDH antibody 6 C5 (Calbiochem). Horseradish peroxidase (HRP) was detected using an enhanced chemiluminescence (ECL) kit (Bio-Rad).

### Cell surface staining and flow cytometry analysis to detect the binding ability of site mutated S proteins to human ACE2

Spike expressor and site-mutated Spike expressor were transfected into 1 × 10^6^ 293 T cells with a weight of 3 μg, respectively. Cells were pre-incubated with 500 nM ACE2-biotyl after 24h post-transfection. ACE2 binding was detected using PE-conjugated streptavidin (Invitrogen). The percentage of ACE2 binding cells (PE+ cells) was determined by gating the living cell population based on viability dye staining (Aqua Vivid, Invitrogen). Samples were acquired on an LSRII cytometer (BD Biosciences, Mississauga, ON, Canada), and data analysis was performed using FlowJo vX.0.7 (Tree Star, Ashland, OR, USA).

### Production and titration of SARS-CoV-2 S pseudoviruses

SARS-CoV-2 pseudotyped viruses were produced as previously described with some modifications [51]. Briefly, 5 × 10^6^ HEK 293 T cells were co-transfected with 6 μg each of pNL4-3. Luc. R -E- and 6 μg recombinant SARS-CoV-2 S plasmids were transfected into HEK 293 T cells using Lipofectamine 3000 Transfection Reagent (Invitrogen) according to the manufacturer’s instructions. The wild-type and mutated S protein pseudotyped viruses in supernatants were harvested 48 hours after transfection, centrifuged, filtered through a 0.45 μm filter, and stored at −80 °C. The pMD2.G was co-transfected with the pNL4-3. Luc. R-E- plasmid to package the VSV-G pseudovirus as the control. The titers of the pseudoviruses were calculated by determining the concentration of cytosolic gag p24 by ELISA.

### SARS-CoV-2 S-mediated pseudo viral entry assay

To detect S variant-mediated viral entry, 293 T-ACE2 cells (2 × 10^4^) grown on 96-well plates were infected with the same amount of wild-type S or mutated S pseudovirus. The cells were transferred to fresh DMEM medium 12 hours post-infection. After 48–72 hours post-infection, the 293T-ACE2 cells were lysed with 30 μL lysis buffer (Promega, Madison, WI, USA) to measure the pseudo viral transduction. Relative luminescence units (RLU) of Luc activity was detected using the Luciferase Assay Kit (Promega). All experiments were performed at least three times and expressed as mean ± standard deviation (SD).

### Statistical analysis

Differences in the frequencies of SNPs’ alleles between mild and severe groups were compared using the Student’s t-test (t.test in R 3.6.2, two-sided, unadjusted for multiple comparisons). Effect size (Cohen’s d) of the comparisons was calculated with R lsr v.0.5.

## Results

### Distribution of single nucleotide variations (SNVs) among COVID-19 patients

Meta-transcriptomic sequencing was performed on 94 clinical specimens (55 nasal swabs,11 throat swabs, 21 sputa, and 7 bronchoalveolar lavage fluid samples) of 48 hospitalized patients at the Shenzhen Third People’s Hospital in late January 2020. Among these patients, 23 were severe cases and 25 were mild cases (Table S2). After mapping reads and genome assembly, we constructed 43 completely assembled SARS-CoV-2 genomes and 43 partial genomes. SARS-CoV-2 sequences represented 0.01% to 2.00% of all quality-filtered reads and 0.08% to 8.82% of the non-rRNA reads. 20x sequencing depth can yield more than 50% genome coverage (Figure 1b). These samples have an average sequencing depth of 63.4 on the virus genome (Figure 1a). For each sample, the virus’s average sequencing depth showed a strong correlation with the Ct values of the RT-qPCR performed on extracted RNA (Figure 1c).

**Figure 2.**
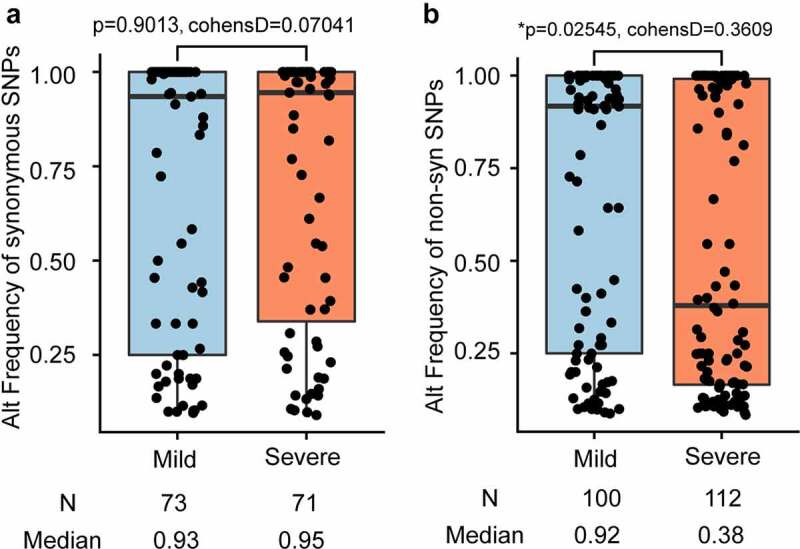
Comparison of SNPs’ alternate frequencies between mild and severe patients

Single nucleotide variants (SNVs) were identified in the SARS-CoV-2 population. To reduce false positives, SNP was filtered based on the following criteria: minor allele was sequenced at least 5 times and had a minimum frequency of 1%. After filtering out low-quality sequencing data, 180 intra-host SNVs of high confidence were identified, including 158 SNPs, 21 deletions, and 1 insertion. The 158 SNPs were identified with high confidence in 41 COVID-19 patients (Table S3). Among the 158 SNPs, 72 are synonymous and 86 nonsynonymous (Table S3). Among these 48 COVID-19 confirmed patients, 7 patients had no identified SNPs, 15 patients have less than 5 SNPs, 9 patients had more than 10 SNPs, and 17 patients has between 5 and 10 SNPs (Figure 1d). Among all identified intra-host SNPs, C8782T and T28,144C were the most commonly shared SNPs, found in more than 24 patients (Figure 1e). The number of SNPs did not differ between mild and severe patients (p-value = 0.2227) (Figure 1d and Figure1f), neither did the alternate frequency of synonymous SNPs (Student’s t-test p-value = 0.9013, (Figure 2a). Nevertheless, the alternate allele frequency of nonsynonymous SNPs in mild patients was notably higher than in severe patients (Student’s t-test p-value = 0.02538, (Figure 2b). We speculate that some non-synonymous mutations probably cause reduced symptoms because competition between virus variants at the population level might create population mosaics of disease characteristics, such as infection fatality rates, transmission, and immune status[52]. With such a small sample size, the results we observe can only reflect one of these symptoms.

**A**. Box plot of synonymous SNPs’ alternate frequencies in mild patients and severe patients. **B**. Box plot of non-synonymous SNPs’ alternate frequencies in mild patients and severe patients. The alternate frequencies of syn and non-syn SNPs possessed by mild and severe patients are compared to each other with the Student’s t-test (t.test in R 3.6.2, two-sided, unadjusted for multiple comparisons).

### Genomic distribution of SNPs reveals that ORF8 can bear alternate alleles with high frequency

**Figure 3.**
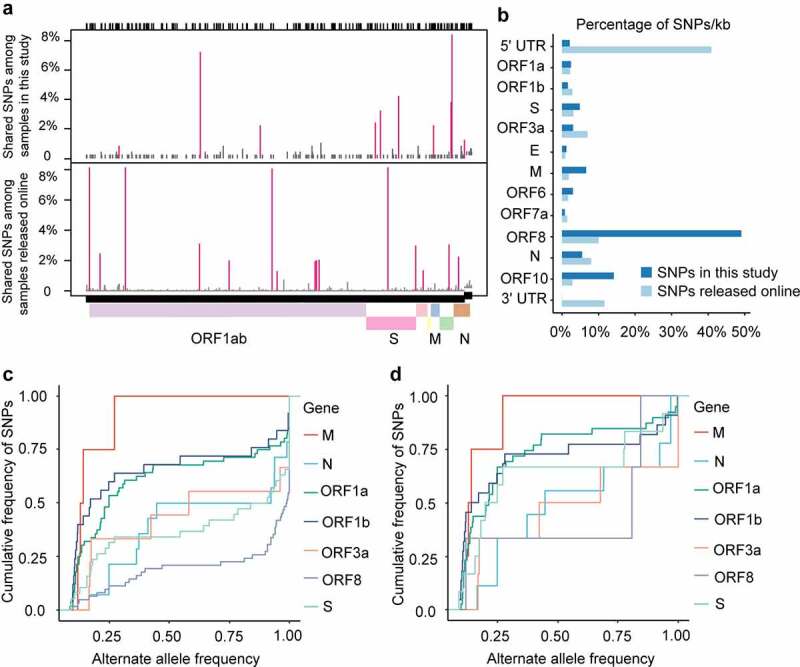
Genomic distribution of SARS-CoV-2 SNPs

We compared the SNPs in our datasets with SNPs from assembled genomes deposited in the GISAID online datasets [53–55] as of April 11, 2020 and found differences in the mutation spectrum (Figure 3a). 5ʹ-UTR is the functional region most significantly enriched with SNPs at an average of 449.06 SNPs per KB compared to an average of 104.43 SNPs per KB across the genome of SARS-CoV-2 based on GISAID SNP dataset (Odd Ratio, OR = 4.30, p-value = 1.89034E-10, (Figure 3b). 3ʹ-UTR had an average of 543.859 GISAID SNPs per KB (Odd Ratio, OR = 5.2078, p-value = 7.15E-11, (Figure 3b) and no significant accumulation of SNPs at the 5ʹ-UTR or 3ʹ-UTR was observed in 94 clinical samples (Figure 3b).

**A**. Location of SNPs detected in this study and GISAID SNPs in the SARS-CoV-2 genome. The y-axis represents the proportion of the sample that contains a certain type of SNP. Positions with coverage < 10x were excluded from the analysis. The shared SNPs of online datasets were analyzed based on 6032 SARS-CoV-2 genome sequences available online as of 11 April 2020 (2019nCoVR, https://bigd.big.ac.cn/ncov). **B**. The percentage of SNPs/kb in ORF regions and non-coding regions for all patients in this study. The value of the y-axis was calculated as the proportion of SNP of a viral gene accounting for the total SNP of the whole viral genome, then normalized by gene length. The calculation formula is “Percentage of SNPs per kb = SNPs of a gene/(Total SNPs of the virus genome×gene length)”. **C**. The cumulative distribution of the alternative allele frequency for the non-synonymous SNPs of different genes among all samples. The y-axis represents the cumulative frequency of occurrence for non-synonymous SNPs from a specific gene, which means that the total frequency of all non-synonymous SNPs in a specific gene is set to 1. According to the value of the alternate frequency of these SNPs, the frequency of occurrence for SNPs with the same alternate frequency are accumulated in ascending order. The x-axis represents the alternative allele frequency of non-synonymous SNPs. **D**. The cumulative distribution of the average alternative allele frequency for each non-synonymous SNP of a specific gene among all samples. To avoid the dominance of certain shared SNPs in the samples, the alternative allele frequency shown here is the mean value of the alternative allele frequencies of the same SNPs among all samples. The y-axis represents the cumulative frequency of occurrence for non-synonymous SNPs from a specific gene. The x-axis represents the alternative allele frequency of SNPs.

**Figure 4.**
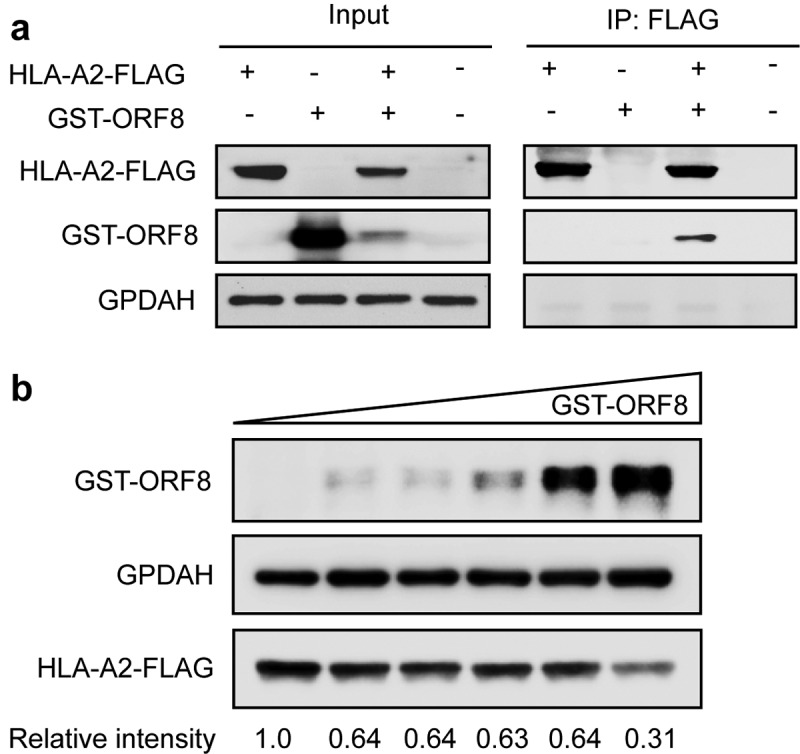
The direct intracellular interaction between ORF8 and MHC I molecular, and ORF8 degrades MHC I in a dose-dependent manner

For virus genomes under positive selection, the ratio of nonsynonymous substitution to synonymous substitution (dN/dS) was greater than 1.0 [3,56]. To test whether the genes of SARS-CoV-2 underwent positive selection during host–virus interaction, we performed positive selection analysis in each gene of the SARS-CoV-2 base on the SNP data in each sample to construct the alternate sequence for each gene using MN908947.3 as the reference. Due to the small sample size, few SNPs in ORF6, ORF7a, E, and a series of NSP genes were identified, so the analysis is not reliable. None of the other genes (M, N, S, ORF3, ORF8, or ORF10) were subjected to significant positive selection according to the likelihood ratio test of positive selection performed by comparing M7 (beta) and M8 (beta&ω) models using PAML4.9 (Table 1). We detected amino acid residues under positive selection for these genes. The G251V of ORF3a used to determine virus V clade[15], was identified as a positively selected site by using the Bayes empirical Bayes (BEB) procedure (Table 1). Furthermore, alleles under positive selection can reach a higher allele frequency in the population than alleles selected against. Thus, a shift of the Alternative Allele Frequency Spectrum (AAFS) of all variants in a particular gene toward higher frequency indicates a positive selective pressure on the gene as a whole. To seek genetic signatures of adaptation of SARS-CoV-2 to the host’s intracellular environment, we examined the AAFS for all SARS-CoV-2 genes (Figure 3c and Figure 3d). All the AAFS for SNPs of the ORF8 gene showed a drastic shift toward higher frequency. The function of the ORF8 gene was largely unclear until recently. An unpublished study showed that the ORF8 protein expressed by SARS-CoV-2 could disrupt the antigen presentation of virus-infected cells by binding directly to the MHC-I molecules and induce the degradation of MHC-I by lysosomes[57]. Here, Co-IP proves the direct intracellular interaction between ORF8 and HLA-A2 (Figure 4a). With the increase of ORF8 expression, the intracellular concentration of HLA-A2 decreased (Figure 4b). It is tempting to speculate that the high variation and positive selection in the viral ORF8 gene may be linked to the extraordinary diversity of human MHC-I genes. ORF8 represents the rapid adaptation of the SARS-CoV-2 genome to the host immune environment.

**Table 1.** Positive selection analysis in the genes of SARS-CoV-2 based on the dataset in this study

Gene	P value (M7:M8)	Positive selected sites with P ≥ 0.95	dN/dS of genes calculated by DnaSP_P
M	0.999997		0.23845
N	0.999999		0.378859
S	0.2919166		0.438138
ORF3a	0.1592752	45 W 49 G 50 V 80 V 89 T 251 G	NA
ORF8	0.2153693		0.362924
ORF10	0.999987		0.101222

**A**. ORF8 was co-immunoprecipitated with the overexpressed HLA-A2. HEK293T Cells were transfected with HLA-A2-FLAG expressing plasmid together with ORF8-GST expressing plasmid or vector for 24 hours before harvest. Cells were collected for co-IP with the anti-FLAG-tag beads. An anti-GPDAH antibody was used as a loading control. A representative result for three independent experiments displayed. **B**. Dose-dependent decreasing of HLA-A by ORF8. HEK293T cell was co-transfected with 0.5 μg pCMV3-HLA-A-Flag plasmid and pCMV-GST-ORF8 of 0, 0.25, 0.5, 1.0, 1.5, and 2.0 μg for 24 hours before harvest. Then equal cell lysis was used for western blotting analysis to detect the expression level of HLA-A. An anti-GPDAH antibody was used as a loading control. A representative result for three independent experiments displayed.

The allele composition and frequency of each SNP site was also analyzed. Among 12 single nucleotide polymorphisms types, A > G, G > A, T > C, and G > T were most common. The ratio of C > T was 40% in our SNPs datasets, which is lower than 50% C > T for GISAID SNPs datasets but similar to 40% C > T iSNV for Houston samples in a recent survey [58] (Figure S1). In humans and many other species, the rate of C > T substitutions is higher than that of other types of substitutions, as methylated Cytosine (C) can be replaced by thymine (T) during DNA replication if the amino group is removed from the methylated cytosine. However, unlike in humans, where the majority of the C > T substitutions occur on the canonical methylated CpG sites, C > T substitutions occur more on CpA, CpT, and CpC sites than on CpG sites in SARS-CoV-2, suggesting a potential distinct mechanism underlying the methylation of the virus genome[59].

### Dynamic of the quasispecies component in SARS-CoV-2 during the period of symptom onset

**Figure 5.**
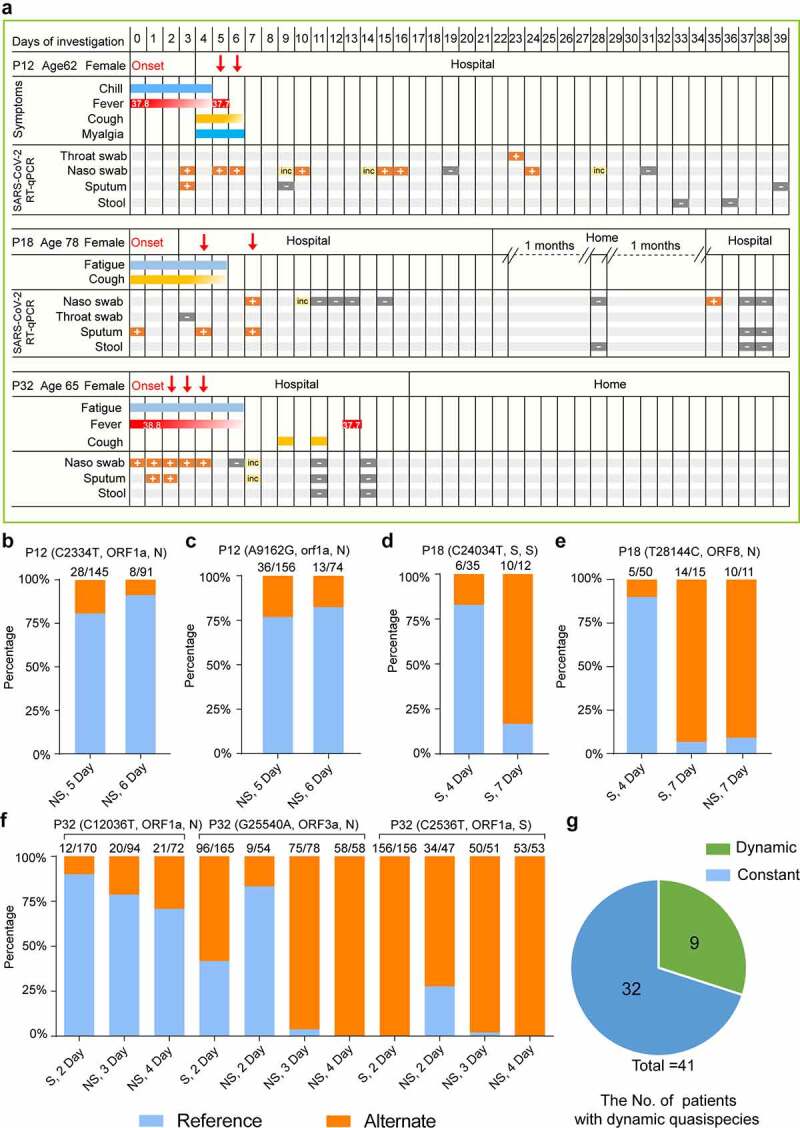
Dynamic of quasispecies’ composition identified in three COVID-19 patients

Analyzing the allele frequency of viral intra-host SNPs at different sampling sites and time points (mainly sputum, nasal swabs) from an individual might help us observe the spatial and temporal dynamics of intra-host variations of SARS-CoV-2 during infection (Figure 5). However, our sampling time point is too close. Only 9 (22%) patients were detected with varying intra-host SNP frequency over time among all patients (Figure 5g and Table S3). For instance, the alternate frequency of SNPs (G25,540A, C2536T, and C12,036T) in the nasal swabs of Patient 32 increased quickly from the second day to the fourth day after symptom onset. G25,540A substitution leads to V50I mutation in ORF3a, C12,036T substitution leads to A3924V mutation in ORF1ab and Nsp3, and the substituted bases eventually took over (figure 5f). From the fourth day to the seventh day after symptom onset, the alternate frequency of C24,034T and T28,144C substitutions in Patient 18 was significantly increased (Figure 5d and Figure 5e). C24,034T mutation leads to synonymous mutation at S protein, T28,144C leads to L84S mutation in ORF8, and 84S eventually dominated the upper respiratory tract of Patient 18 (Figure 5e). Moreover, the alternate frequency of C2334T(A690V, ORF1ab, and Nsp2) and A9162G (N2966S, orf1ab, and Nsp4) of Patient 12 decreased from day 5 to day 6 after symptom onset (Figure 5b and Figure 5c). These data suggest the occurrence of purifying selection of C2334T (A690V, ORF1ab, and Nsp2)/A9162G (N2966S, ORF1ab, and Nsp4) in Patient 12, and positive selection on C12,036T (A3924V, ORF1ab, and Nsp3)/G25,540A (V50I, ORF3a) in Patient 32 and T28,144C (L84S, ORF8) in Patient 18.

**A**. Symptoms and results of RT-qPCR testing for SARS-CoV-2 of the three COVID-19 patients (P12, P18, and P32) by day of the investigation. Gradient shading indicates an unclear period of symptom onset from the patient report. RT-qPCR means Real-time quantitative PCR, inc means inconclusive result. SARS-CoV-2, severe acute respiratory syndrome coronavirus 2. The sampling dates are denoted by red arrows. **B, C**. Allele frequency of C23,34T(**B**), A9162G(**C**) substitutions in nasal swabs collected from Patient 12 (P12) by time course. **D, E**. Allele frequency of C24,034T(**D**), T28,144C (**E**) substitutions in sputum and nasal swabs collected at time points from Patient 18 (P18). **F**. Allele frequency of G24,430A, C2536T, C12,036T substitutions in different types of samples (sputum and nasal swab) collected from Patient 32 (P32). Allele frequency of G24,430A, C2536T, and C12,036T substitutions in nasal swabs collected from Patient 32 at different time points.The allele frequency was analyzed using Wuhan-hu-1(MN908947.3) as a reference. The alternate allele depth/total depth for each sample is shown up the bar. **G**. The percentage of patients with dynamic quasispecies diversity.

To study the correlation of mutation with the disease severity, we checked the patients’ clinical records and found the sampling date almost followed the onset date. As shown in Figure 5a, samples were collected 5 days, 4 days, and 2 days after symptom onset date for Patients12, 18, and 32, respectively, suggesting that the virus begins to evolve early during the onset of symptoms. We hypothesized that the virus evolved rapidly in the acute phase of infection due to the human body’s intense immune response. It is important to note that although Patients 12 and 18 were classified as mild, the RT-qPCR results for SARS-CoV-2 remained positive for a long time. For example, Patient 18 tested positive even after two months of home isolation after discharge. We hypothesize that mutations in the virus resulted in its persistence within Patients 12 and 18 (Table S2 and S3).

### Co-occurrence of SNPs was associated with the severity of disease

**Figure 6.**
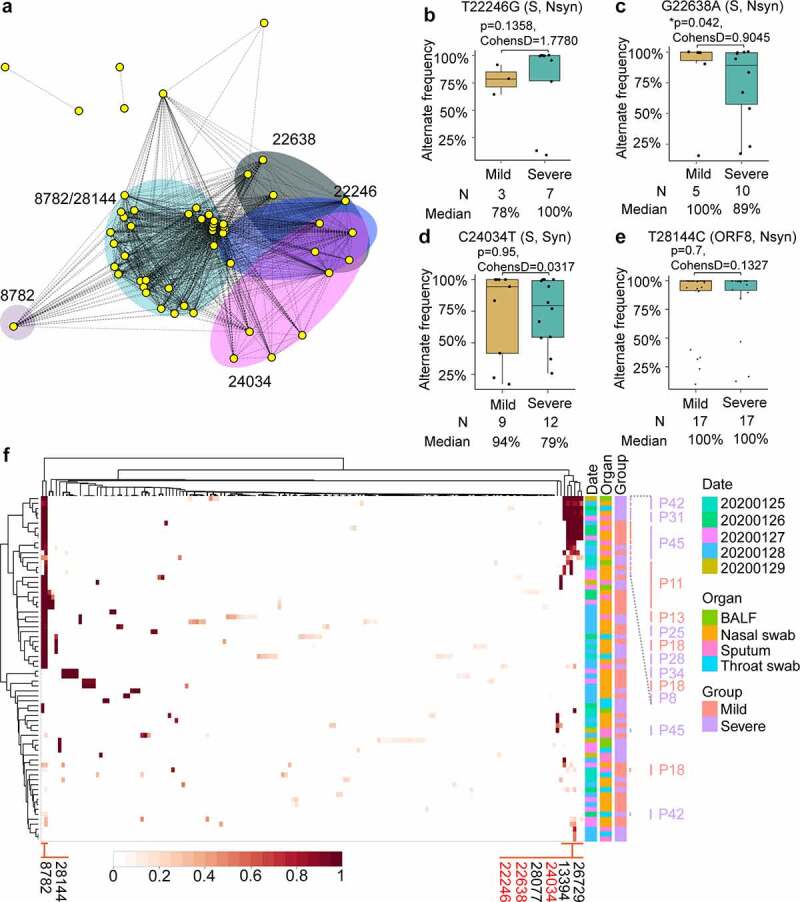
Co-occurring SNPs were found in S glycoprotein among COVID-19 patients

To explore the dominant SNPs in the study population, shared SNPs of the virus were further analyzed. A SNP network connecting patients with shared SNPs was constructed. In this network, nearly all patients are connected by at least one shared SNP and the most abundant five SNPs formed four major clusters (Figure 6a). SNPs at 8782(C-T) and 28,144(T-C) coexisted in the patients, forming the largest cluster. The clustering heatmap of allele frequency of different samples collected from mild and severe patients also revealed that SNPs at 8782 and 28,144 frequently occur together and are the most common variants among samples (figure 6f). Interestingly, SNPs at sites 22,246(T-G), 22,638(G-A), 28,077(G-C), 24,034(C-T), 13,394(A-G), and 26,729(T-C) with high alternating frequencies can be detected simultaneously in a sample (figure 6f). In this cluster, 7 (70%) patients came from the severe group and 3 (30%) patients were in the mild group. This cluster of patients did not belong to the same family or community, nor did they have direct contact with each other, suggesting that the variations at these loci were randomly distributed and dominant in the population. Among the most abundant SNPs, SNPs at sites 22,246, 22,638, and 24,034 were all distributed in the S glycoprotein region and Site 28,144 was located at ORF8. The T-to-G substitution at site 22,246 leads to Asp228Glu amino-acid substitution of S protein; the G-to-A nucleotide substitution at site 22,638 causes a Ser359Asn amino acid substitution of S protein; and the C-to-T substitution at 28,144 leads to the Leu84Ser amino-acid substitution of ORF8. In the early phases of the epidemic, the L84S mutation in ORF8 resulted in two subgroups of the virus[60].

**A**. Network analysis of patients with shared SNP sites. Nodes denote patients, and edges indicate that two patients have the same SNP sites. Clusters of the network are highlighted by colored shading, and the shared SNP sites leading to the clusters are shown. **B**. Box plot of the alternate frequency of T22,246G SNP in mild and severe patients. The location of SNP in the genome was shown. S, S glycoprotein. Nsyn, Non-synonymous. The reference was MN908947.3. **C**. The alternate frequency of G22,638A SNP in mild and severe patients. S, S glycoprotein. Nsyn, Non-synonymous. **D**. The alternate frequency of C24,034T SNP in mild and severe patients. S, S glycoprotein. Syn, synonymous. **E**. The alternate frequency of T28, 144C SNP in mild and severe patients. ORF8, T28,144C SNP located in ORF8 region of SARS-CoV-2 genome. Boxes represent the interquartile range (IQR) between the first and third quartiles (25th and 75th percentiles, respectively). Lines inside denote the median. T-test was used to compare alternate frequencies of SNPs between the mild and severe groups. **F**. The cluster heatmap of SNP in different samples collected from mild and severe patients by time point. Sampling date, organ, and population group are shown by a different color in right-side boxes; the samples with shared SNPs and relative patients were highlighted. The x-axis represents the identified SARS-CoV-2 SNPs in this study, which clustered according to its alternate frequency. Alternate frequencies of SNPs are showed with a gradient color marker. White represents the SNP alternate frequency value of 0; the genomic regions failed to pass our criteria used in identifying SNPs. Dark red indicates an alternate frequency approaching 1. The y-axis represents samples clustered by different mutation types. P means patient. BALF, bronchoalveolar lavage fluid.

Subsequently, whether the allele frequency of these dominant SNPs was associated with disease severity was analyzed. The alternate frequency of SNP at sites 22,246, 22,638, 24,034, and 28,144 was analyzed in patients with different severity. The alternate frequency distribution of SNP at sites 22,638 was statistically lower in severe patients than in mild patients (p = 0.042, cohensD = 0.9045, (Figure 6c). No differences were found for the alternate frequency of SNPs at sites 22,246, 24,034, and 28,144 between the mild and severe groups (p = 0.1358, 0.95, and 0.7 for sites 22,246, 24,034, and 28,144, respectively, (Figure 6b, Figure 6d, and Figure 6e). The S359N (G22,638A) mutation on the S protein seems to be more likely to appear in mild patients based on our small sample. However, we need more samples and experiments to determine whether the mutation of S359N will affect the fitness of the virus in the host.

### Site 359 of S protein does not affect its binding to ACE2 but is a candidate epitope for CTL response

**Figure 7.**
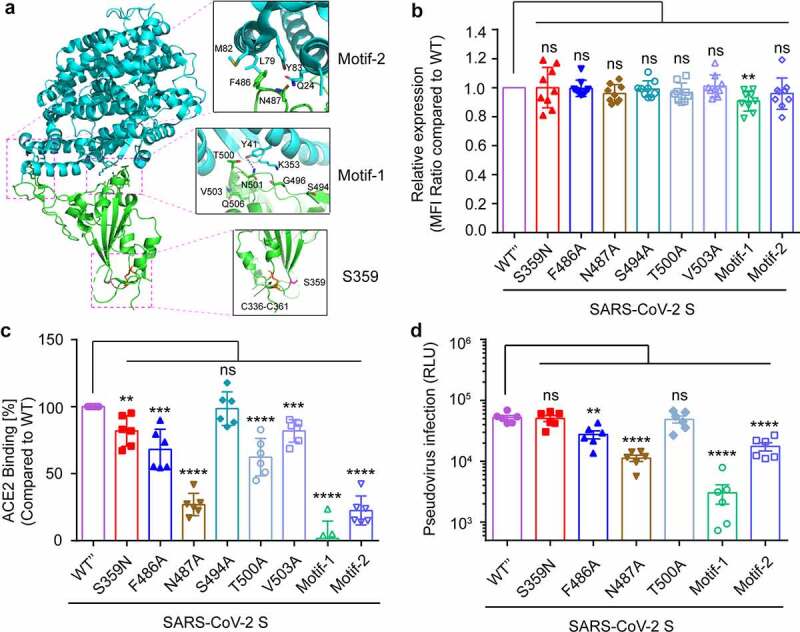
Functional and structural insights into S359N variant of SARS-CoV-2 S glycoprotein

To investigate the effect of intra-host genomic variations of SARS-CoV-2 on its biological function and pathogenicity, the function of SNPs with high alternate frequency was further analyzed. According to the high-resolution cryo-EM structure information of SARS-CoV-2 S glycoprotein (PDB: 6LZG), the receptor-binding domain (RBD) interacts with the human ACE2 receptor to mediate the binding of the virus to the target cell. The receptor-binding motif (RBM) in RBD forms the interface between S glycoprotein and human ACE2 [61,62]. The Ser359Asn substitution was located in the RBD domain but outside the RBM region (Figure 7a), implicating amino acid 359 does not directly interact with ACE2[63]. As the region outside the RBM also plays an important role in maintaining the RBD’s structural stability[64], whether the S359N mutation altered the virulence of the virus and its fitness in the host was explored. A series of s glycoprotein site mutations expression vectors were constructed, then transfect 293 T cell (Figure 7b) and ACE2 binding ability to mutated S proteins were detected by cell surface staining and flow cytometry analysis. Compared to the F486A and N487A in RBM motif-2 and T500A in RBM motif-1, the S359N mutation led to a slight decrease of ACE2-binding (Figure 7c), but this slight change was not enough to affect the efficiency of the pseudovirus infection (Figure 7d) and is not an important site for maintaining ACE2 binding. Recently, a study reported that the top eight neutralizing antibodies maintained their potency against S359N variants[65], so the S359N variant is not an escape mutant from neutralizing antibodies. As in the host, the virus will face cellular immune responses as well as humoral immune responses. Thus, different MHC binding and T cell responses should be considered as triggers for viral mutations. The peptides of S protein for binding to most frequent MHC class I (A&B) alleles in Europe, Asia, and Africa were analyzed (Table S5). The peptides with S359 inside can bind to the HLA-A30 allele (consensus_percentile_ranks<1) and S359 is an HLA-A30 restricted epitope.

**A**. Overall structure of SARS-CoV-2 RBD bound to human ACE2. The interface of RBD and ACEs are displayed as RBM motif-1 and RBM motif-2. The amino acid S359 and amino acids in the receptor-binding motif are shown as sticks. **B**. Detection of the expression of site-mutated S glycoprotein in 293 T cell line. **C**. Cell staining and flow cytometry analysis of site-mutated S glycoprotein binding to ACE2. The experiment was repeated three times, and similar results were obtained. **D**. Detection site-mutation derived SARS-CoV-2 pseudovirus infection. The data are expressed as mean relative luciferase units (RLU) ± standard deviation (SD) of 4 parallel wells in 96-well culture plates. The relative luminescence units (RLU) detected 72 hours post-infection (hpi). The experiment was repeated three times and similar results were obtained.

### L84S mutation is an candidate for HLA-A02 restricted CTL epitope

ORF8 directly interacts with human MHC I molecular. The high frequency of SNP28,144 (L84S) was widespread and detected in most samples in this study, suggesting that this site may be selected (Figure 3c and Figure 3d, Table S3). It is also predicted that peptides of ORF8 bind to the MHC I molecule, based on the IEDB analysis resource using a consensus method[66]. The wild-type peptides containing site L84 can bind to HLA-A*02:06, common in Asian populations. HLA-A*02:06 accommodates the wild-type, but not the SARS-CoV-2 ORF8 (L84S) variant (Table 2). To show the accuracy of these predictions, the literature was reviewed for HA peptides of H5N1 influenza viruses that bind to HLA-A*02:01, which have been experimentally verified as HLA-A*02:01-restricted peptides for H5 HA peptides[67]. The ORF8 L84S variants changed peptide binding to MHC class I alleles, meaning the site 84 of ORF8 may be a potentially cytotoxic T-lymphocyte epitope.

**Table 2.** ORF8 L84S variants and differential peptide binding to the HLA-A allele

Protein	Peptide	Rank wt	Rank mut	Allele
HA	LLLAIVSLV	0.3		HLA-A*02:01
HA	GILGFVFTL	0.8		HLA-A*02:01
HA	KLYQNPTTYI	0.47		HLA-A*02:01
HA	VLLLAIVSL	0.8		HLA-A*02:01
HA	RLYQNPTTYI	0.9		HLA-A*02:01
ORF8	NYTVSC**L**(**S**)PF	0.22	0.3	HLA-A*23:01
ORF8	NYTVSC**L**(**S**)PF	0.33	0.7	HLA-A*24:02
ORF8	YTVSC**L**(**S**)PFTI	0.52	1.29	HLA-A*02:06
ORF8	TVSC**L**(**S**)PFTI	0.9	1.1	HLA-A*68:02
ORF8	IGNYTVSC**L**(**S**)PF	1.0	1.5	HLA-A*23:01

A lower rank value designates better MHC class I; we were using the consensus percentile rank <1.0 as the cutoff value for binding. HLA-A*02:06, A*68:02, and A*23:01 accommodate the wild-type ORF8 peptides, yet not the variant L84S. The HLA-A*02:01 restricted H5N1 Flu A hemagglutinin epitopes show strong binding to HLA-A*02:01, which is predicted by the same method.

## Discussion

RNA viruses replicate in-vivo as a quasispecies, a dynamic distribution of divergent but closely related genomes subjected to a continuous process of genetic variation, competition, and selection[3]. This genomic heterogeneity confers a remarkable advantage to the viral population allowing for a rapid adaptation to a changing environment. Although SNVs have been identified in the SARS-CoV-2 genome and some of them contribute to viral pathogenicity[68], more viral genomic data from clinical specimens needs to be analyzed in order to study intra-host SNV dynamics, which may directly reflect virus-host interaction.

This study addresses this gap by using metatranscriptomic sequencing to find SNVs from 94 sequenced clinical samples of 48 COVID-19 patients with varying disease severity. One hundred fifty-eight SNPs were identified with high confidence in 70 samples of 41 COVID-19 patients after removing the low-quality reads and samples with low coverage and less than 10-fold sequencing depth. The positive selection analysis of genes in the SARS-CoV-2 genome based on our datasets did not result in any genes being subjected to positive selection. Our result is different from an earlier study, which reported that ORF3a and ORF8 were under positive selection and exhibited higher dN/dS ratios than other genes at the level of the individual gene [69], but this may be due to the small sample size of this study. As no synonymous SNP in ORF3 were detected, it is difficult to conclude a more widely representative dN/dS ratio at the level of individual genes. According to our dataset, ORF3a has a positively selected amino acid G251V, consistent with the previous literature[69]. Additionally, in this study, ORF8 seemed to be more tolerant of some high-frequency alternate allele. It is worth noting that 84(L > S) is a high-frequency non-synonymous mutation found on ORF8 in our data set, which is contrary to an earlier survey that found L84 lineage is more prevalent than S84 lineage[60]. The function of SARS-CoV-2 ORF8 has not yet been clarified, though the SARS-CoV-2 382-nt deletion variant with truncated ORF8 was detected in Singapore and Taiwan [70,71]. The SARS-CoV-2 382-nt deletion viruses showed significantly higher replicative fitness in vitro than the wild type[70]. In one recent report, ORF8 was determined to be a protein secreted by infected cells into serum and is highly immunogenic in COVID-19 patients [72]. Thus, we predict ORF8 peptides bind to the MHC class I allele. Some specific MHC class I alleles accommodate L84 peptides but not S84 peptides (Table 2). Under host selective pressure, ORF8 deletions or site mutations may occur to evade antibody neutralization and cellular immunity, allowing SARS-CoV-2 to escape clearance from the host’s immune system.

The correlation between viral quasispecies evolution and pathogenicity has been confirmed in prior studies [68]. Our analysis of shared SNPs among patients with different severity suggest that mild patients had higher alternate frequency SNPs at 22,638 (S359N). Because of the small sample size of this study, it is difficult to conclude if the S359N mutation caused reduced symptoms in patients. However, S359N, a common mutation in the population was determined. The reason for the emergence of this S359N variant is not clear. In a recent survey, eight top NAbs maintained their potency against the S359N variant [65], suggesting that S359 might not be the target of humoral immunity. The TepiTool was used to predict T cell epitope candidates for S protein of SARS-CoV-2, which revealed peptide (RISNCVADY) with site S359 is an HLA-A*30 restricted T cell epitope (Table S5). Whether these circulating SARS-CoV-2 variants are associated with T-cell responses or neutralizaing antibodies still needs to be clarified. By comparing SNPs’ allele frequencies in samples collected from Patients 18 and 32 at similar time points, we found a novel intra-host viral profile of SARS-CoV-2. As seen in Patient 18, the mutation S84 (28,144 C) on ORF8 eventually replaced L84 (28,144 T) as the dominant variant in quasispecies in the patient’s sputum. This is reminiscent of rapidly changing viral lineages during acute HIV-1 infection, namely the rapid replacement of the major transmitted/founder lineage by a minor transmitted/founder lineage[73]. This phenomenon may be due to the interplay between viral and host factors. The type I IFNs and amount of antibodies in serum changed dramatically at symptom onset [73], which will exert selective pressure on the virus genome.

Studies on the quasispecies diversity of other RNA viruses have found more abundant intra-host genetic variations. For example, metatranscriptomic sequencing results of HIV-1 in the blood and female genital tract have identified 77 iSNVs in an individual [74]. Research on intra-host dynamics of the Ebola virus during 2014 identified 710 iSNVs in 135 EBOV samples[56]. Fewer SNVs were identified in SARS-CoV-2 positive samples from this study, which could in part be attributable to lower sequencing depth (due to the low viral load in the sample), but also likely reflects some true loss of diversity. As a β-coronavirus, SARS-CoV-2 encodes an RNA dependent RNA polymerases (RdRPs) with a high-fidelity nucleotide incorporation ability[75]. Its CoV nonstructural protein14 (*nsp*14) also encodes 3ʹ-to-5ʹ exoribonuclease activity (ExoN), which performs a proofreading function and is required for high-fidelity replication[75]. This would provide some evidence for the low diversity of the intra-host population for SARS-CoV-2 to some extent.

In conclusion, frequent sampling and metatranscriptomic sequencing was utilized to study SARS-CoV-2 populations present in upper and lower respiratory tracts of patients with different severity. Rapid and dramatic changes in quasispecies diversity were observed, providing new insights into the intra-host evolution of SARS-CoV-2. However, our current research has great limitations due to the small sample size. More samples are needed to investigate the intra-host genomic diversity of SARS-CoV-2. Furthermore, more experiments should be carried out to explore the host immune system’s influence on virus evolution.

## Supplementary Material

Supplemental Material

## References

[1] Kunkel TA. Exonucleolytic proofreading. Cell. 1988;53(6):837–840. 2838173 10.1016/s0092-8674(88)90189-4

[2] Vaughan G, Goncalves Rossi LM, Forbi JC, et al. Hepatitis A virus: host interactions, molecular epidemiology and evolution. Infect Genet Evol. 2014;21:227–243. 24200587 10.1016/j.meegid.2013.10.023

[3] Domingo E, Sheldon J, Perales C. Viral quasispecies evolution. Microbiol Mol Biol Rev. 2012;76:159–216. 22688811 10.1128/MMBR.05023-11 PMC3372249

[4] Nowak MA. What is a quasispecies? Trends Ecol Evol. 1992;7(4):118–121. 21235976 10.1016/0169-5347(92)90145-2

[5] Barik S, Das S, Vikalo H. QSdpR: viral quasispecies reconstruction via correlation clustering. Genomics. 2018;110(6):375–381. 29268961 10.1016/j.ygeno.2017.12.007

[6] Xu D, Zhang Z, Wang FS. SARS-associated coronavirus quasispecies in individual patients. N Engl J Med. 2004;350(13):1366–1367. 15044654 10.1056/NEJMc032421

[7] Park D, Huh HJ, Kim YJ, et al. Analysis of intrapatient heterogeneity uncovers the microevolution of middle east respiratory syndrome coronavirus. Cold Spring Harb Mol Case Stud. 2016;2(6):a001214. 27900364 10.1101/mcs.a001214 PMC5111008

[8] Zhou P, Yang X, Wang X, et al. A pneumonia outbreak associated with a new coronavirus of probable bat origin. Nature. 2020;579(7798):270–273. 32015507 10.1038/s41586-020-2012-7 PMC7095418

[9] Li X, Giorgi EE, Marichannegowda MH, et al. Emergence of SARS-CoV-2 through recombination and strong purifying selection. Sci Adv. 2020;6(27):eabb9153. 32937441 10.1126/sciadv.abb9153 PMC7458444

[10] Forster P, Forster L, Renfrew C, et al. Phylogenetic network analysis of SARS-CoV-2 genomes. Proc Natl Acad Sci U S A. 2020;117(17):9241–9243. 32269081 10.1073/pnas.2004999117 PMC7196762

[11] Van Dorp L, Acman M, Richard D, et al. Emergence of genomic diversity and recurrent mutations in SARS-CoV-2. Infect Genet Evol. 2020;83:104351. 32387564 10.1016/j.meegid.2020.104351 PMC7199730

[12] Rambaut A, Loman N, Pybus O, et al. Preliminary genomic characterisation of an emergent SARS-CoV-2 lineage in the UK defined by a novel set of spike mutations. Virological org. 2020.

[13] Domingo-Calap P, Schubert B, Joly M, et al. An unusually high substitution rate in transplant-associated BK polyomavirus in vivo is further concentrated in HLA-C-bound viral peptides. PLoS Pathog. 2018;14(10):e1007368. 30335851 10.1371/journal.ppat.1007368 PMC6207329

[14] Holmes EC, Dudas G, Rambaut A, et al. The evolution of Ebola virus: insights from the 2013–2016 epidemic. Nature. 2016;538(7624):193–200. 27734858 10.1038/nature19790 PMC5580494

[15] Gámbaro F, Behillil S, Baidaliuk A, et al. Introductions and early spread of SARS-CoV-2 in France. Euro Surveill. 2020;25(26):2001200. 10.2807/1560-7917.ES.2020.25.26.2001200 PMC7346363 32643599

[16] Rambaut A, Holmes EC, Á O, et al. A dynamic nomenclature proposal for SARS-CoV-2 lineages to assist genomic epidemiology. Nat Microbiol. 2020;5(11):1403–1407. 32669681 10.1038/s41564-020-0770-5 PMC7610519

[17] Kupferschmidt K. Fast-spreading U.K. virus variant raises alarms. Science. 2021;371(6524):9–10. 33384355 10.1126/science.371.6524.9

[18] Santos JC, Passos GA. The high infectivity of SARS-CoV-2 B.1.1.7 is associated with increased interaction force between Spike-ACE2 caused by the viral N501Y mutation. bioRxiv. 2020. doi: 10.1101/2020.12.29.424708

[19] Gaudieri S, Rauch A, Park LP, et al. Evidence of viral adaptation to HLA class I-restricted immune pressure in chronic hepatitis C virus infection. J Virol. 2006;80(22):11094–11104. 17071929 10.1128/JVI.00912-06 PMC1642167

[20] Cuypers L, Li G, Libin P, et al. Selective pressure in hepatitis C virus genotypes 1–6: significance for direct-acting antiviral treatment and drug resistance. Viruses. 2015;7(9):5018–5039. 26389941 10.3390/v7092857 PMC4584301

[21] Ghadimi-Moghadam A, Haghani M, Bevelacqua JJ, et al. COVID-19 tragic pandemic: concerns over unintentional “directed accelerated evolution” of novel coronavirus (SARS-CoV-2) and introducing a modified treatment method for ARDS. J Biomed Phys Eng. 2020;10(2):241–246. 32337192 10.31661/jbpe.v0i0.2003-1085 PMC7166223

[22] Azkur AK, Akdis M, Azkur D, et al. Immune response to SARS-CoV-2 and mechanisms of immunopathological changes in COVID-19. Allergy. 2020;75(7):1564–1581. 32396996 10.1111/all.14364 PMC7272948

[23] Xu X, Gao X. Immunological responses against SARS-coronavirus infection in humans. Cell Mol Immunol. 2004;1(2):119–122. 16212898

[24] Hodgins B, Yam KK, Winter K, et al. A single intramuscular dose of a plant-made virus-like particle vaccine elicits a balanced humoral and cellular response and protects young and aged mice from influenza H1N1 virus challenge despite a modest/absent humoral response. Clin Vaccine Immunol. 2017;24(12):e00273–17. 29021303 10.1128/CVI.00273-17 PMC5717192

[25] Da Costa XJ, Brockman MA, Alicot E, et al. Humoral response to herpes simplex virus is complement-dependent. Proc Natl Acad Sci USA, 1999; 96(22):12708–12712. 10.1073/pnas.96.22.12708 PMC23060 10535987

[26] Veron P, Leborgne C, Monteilhet V, et al. Humoral and cellular capsid-specific immune responses to adeno-associated virus type 1 in randomized healthy donors. J Immunol. 2012;188(12):6418–6424. 22593612 10.4049/jimmunol.1200620

[27] Rogers TF, Zhao F, Huang D, et al. Isolation of potent SARS-CoV-2 neutralizing antibodies and protection from disease in a small animal model. Science. 2020;369(6506):956–963. 32540903 10.1126/science.abc7520 PMC7299280

[28] Long QX, Liu BZ, Deng HJ, et al. Antibody responses to SARS-CoV-2 in patients with COVID-19. Nat Med. 2020;26(6):845–848. 32350462 10.1038/s41591-020-0897-1

[29] Amanat F, Stadlbauer D, Strohmeier S, et al. A serological assay to detect SARS-CoV-2 seroconversion in humans. Nat Med. 2020;26(7):1033–1036. 32398876 10.1038/s41591-020-0913-5 PMC8183627

[30] Wu F, Wang A, Liu M, et al. Neutralizing antibody responses to SARS-CoV-2 in a COVID-19 recovered patient cohort and their implications. medRxiv. 2020; doi: 10.1101/2020.03.30.20047365.

[31] Ni L, Ye F, Cheng M-L, et al. Detection of SARS-CoV-2-Specific humoral and cellular immunity in COVID-19 convalescent individuals. Immunity. 2020;52(6):971–977. 10.1016/j.immuni.2020.04.023 PMC7196424 32413330

[32] Baruah V, Bose S. Immunoinformatics-aided identification of T cell and B cell epitopes in the surface glycoprotein of 2019-nCoV. J Med Virol. 2020;92(5):495–500. 32022276 10.1002/jmv.25698 PMC7166505

[33] Draper JL, Hansen LM, Bernick DL, et al. Fallacy of the unique genome: sequence diversity within single helicobacter pylori strains. mBio. 2017;8(1):e02321–16. 28223462 10.1128/mBio.02321-16 PMC5358919

[34] Roedig JV, Rapp E, Höper D, et al. Impact of Host Cell Line Adaptation on Quasispecies Composition and Glycosylation of Influenza A Virus Hemagglutinin. Plos One. 2011;6(12):e27989. 22163276 10.1371/journal.pone.0027989 PMC3233551

[35] Kissi B, Badrane H, Audry L, et al. Dynamics of rabies virus quasispecies during serial passages in heterologous hosts. J Gen Virol. 1999;80(8):2041–2050. 10466802 10.1099/0022-1317-80-8-2041

[36] Ke R, Zitzmann C, Ribeiro RM, et al. Kinetics of SARS-CoV-2 infection in the human upper and lower respiratory tracts and their relationship with infectiousness. medRxiv. 2020. 2020.09.25.20201772. 10.1101/2020.09.25.20201772

[37] Branchett WJ, Lloyd CM. Regulatory cytokine function in the respiratory tract. Mucosal Immunol. 2019;12(3):589–600. 30874596 10.1038/s41385-019-0158-0 PMC7051906

[38] Zuercher AW, Jiang H-Q, Thurnheer MC, et al. Distinct mechanisms for cross-protection of the upper versus lower respiratory tract through intestinal priming. J Immunol. 2002;169(7):3920–3925. 12244191 10.4049/jimmunol.169.7.3920

[39] Kechin A, Boyarskikh U, Kel A, et al. cutPrimers: a new tool for accurate cutting of primers from reads of targeted next generation sequencing. J Comput Biol. 2017;24(11):1138–1143. 28715235 10.1089/cmb.2017.0096

[40] Li H, Durbin R. Fast and accurate short read alignment with Burrows–Wheeler transform. Bioinformatics. 2009;25(14):1754–1760. 19451168 10.1093/bioinformatics/btp324 PMC2705234

[41] Li H, Handsaker B, Wysoker A, et al. The sequence alignment/map format and SAMtools. Bioinformatics. 2009;25(16):2078–2079. 19505943 10.1093/bioinformatics/btp352 PMC2723002

[42] McKenna A, Hanna M, Banks E, et al. The genome analysis toolkit: a mapreduce framework for analyzing next-generation DNA sequencing data. Genome Res. 2010;20(9):1297–1303. 20644199 10.1101/gr.107524.110 PMC2928508

[43] Tamura K, Peterson D, Peterson N, et al. MEGA5: molecular evolutionary genetics analysis using maximum likelihood, evolutionary distance, and maximum parsimony methods. Mol Biol Evol. 2011;28(10):2731–2739. 21546353 10.1093/molbev/msr121 PMC3203626

[44] Price MN, Dehal PS, Arkin AP. FastTree: computing large minimum evolution trees with profiles instead of a distance matrix. Mol Biol Evol. 2009;26(7):1641–1650. 19377059 10.1093/molbev/msp077 PMC2693737

[45] Yang Z. PAML 4: phylogenetic analysis by maximum likelihood. Mol Biol Evol. 2007;24(8):1586–1591. 17483113 10.1093/molbev/msm088

[46] Yang Z, Nielsen R, Goldman N, et al. Codon-substitution models for heterogeneous selection pressure at amino acid sites. Genetics. 2000;155(1):431–449. 10790415 10.1093/genetics/155.1.431 PMC1461088

[47] Nei M, Gojobori T. Simple methods for estimating the numbers of synonymous and nonsynonymous nucleotide substitutions. Mol Biol Evol. 1986;3(5):418–426. 3444411 10.1093/oxfordjournals.molbev.a040410

[48] Librado P, Rozas J. DnaSP v5: a software for comprehensive analysis of DNA polymorphism data. Bioinformatics. 2009;25(11):1451–1452. 19346325 10.1093/bioinformatics/btp187

[49] Kassambara A. ggplot2: guide to create beautiful graphics in R. Create Space Independent Publishing Platform; 2016; 1:236.

[50] Shannon P, Markiel A, Ozier O, et al. Cytoscape: a software environment for integrated models of biomolecular interaction networks. Genome Res. 2003;13(11):2498–2504. 14597658 10.1101/gr.1239303 PMC403769

[51] Crawford KH, Eguia R, Dingens AS, et al. Protocol and reagents for pseudotyping lentiviral particles with SARS-CoV-2 spike protein for neutralization assays. Viruses. 2020;12(5):513. 10.3390/v12050513 PMC7291041 32384820

[52] Danchin A, Timmis K. SARS-CoV-2 variants: relevance for symptom granularity, epidemiology, immunity (herd, vaccines), virus origin and containment? Environ Microbiol. 2020;22(6):2001–2006. 32367648 10.1111/1462-2920.15053 PMC7267449

[53] Zhao WM, Song SH, Chen ML, et al. The 2019 novel coronavirus resource. Hereditas (Beijing). 2020;42:212–221. 10.16288/j.yczz.20-030 32102777

[54] Song S, Ma L, Zou D, et al. The global landscape of SARS-CoV-2 genomes, variants, and haplotypes in 2019nCoVR. Genomics Proteom Bioinform. 2020 S1672-0229(20)30131-5. 10.1016/j.gpb.2020.09.001 PMC7836967 33704069

[55] Gong Z, Zhu JW, Li CP, et al. An online coronavirus analysis platform from the national genomics data center. Zool Res. 2020;41(6):705–708. 33045776 10.24272/j.issn.2095-8137.2020.065 PMC7671910

[56] Ni M, Chen C, Qian J, et al. Intra-host dynamics of Ebola virus during 2014. Nat Microbiol. 2016;1(11):16151. 27595345 10.1038/nmicrobiol.2016.151

[57] Zhang Y, Zhang J, Chen Y, et al. The ORF8 protein of SARS-CoV-2 mediates immune evasion through potently downregulating MHC-I. bioRxiv. 2020. doi: 10.1101/2020.05.24.111823.

[58] Sapoval N, Mahmoud M, Jochum MD, et al. SARS-CoV-2 genomic diversity and the implications for qRT-PCR diagnostics and transmission. Genome Res. 2021; 31(4):635-644 10.1101/gr.268961.120 PMC8015855 33602693

[59] Robertson KD, Jones PA. DNA methylation: past, present and future directions. Carcinogenesis. 2000;21(3):461–467. 10688866 10.1093/carcin/21.3.461

[60] Tang X, Wu C, Li X, et al. On the origin and continuing evolution of SARS-CoV-2. Natl Sci Rev. 2020;7(6):1012–1023. 10.1093/nsr/nwaa036 PMC7107875 34676127

[61] Wang Q, Zhang Y, Wu L, et al. Structural and functional basis of SARS-CoV-2 entry by using human ACE2. Cell. 2020;181(4):894–904.e9. 32275855 10.1016/j.cell.2020.03.045 PMC7144619

[62] Morse JS, Lalonde T, Xu S, et al. Learning from the past: possible urgent prevention and treatment options for severe acute respiratory infections caused by 2019-nCoV. Chembiochem. 2020;21(5):730–738. 32022370 10.1002/cbic.202000047 PMC7162020

[63] Walls AC, Park YJ, Tortorici MA, et al. Structure, function, and antigenicity of the SARS-CoV-2 spike glycoprotein. Cell. 2020;181(2):281–292.e6. 10.1016/j.cell.2020.02.058 PMC7102599 32155444

[64] Cagliani R, Forni D, Clerici M, et al. Computational inference of selection underlying the evolution of the novel coronavirus, SARS-CoV-2. J Virol. 2020;94(12):e00411-20. 10.1128/JVI.00411-20 PMC7307108 32238584

[65] Baum A, Bo F, Wloga E, et al. Antibody cocktail to SARS-CoV-2 spike protein prevents rapid mutational escape seen with individual antibodies. Science. 2020;369(6506):1014–1018. 32540904 10.1126/science.abd0831 PMC7299283

[66] Paul S, Sidney J, Sette A, et al., TepiTool: A pipeline for computational prediction of T Cell Epitope Candidates. Curr Protoc Immunol. 2016; 114: 18.19.1-18.19.24. 10.1002/cpim.12 PMC4981331 27479659

[67] Sun Y, Liu J, Yang M, et al. Identification and structural definition of H5-specific CTL epitopes restricted by HLA-A*0201 derived from the H5N1 subtype of influenza A viruses. J Gen Virol. 2010;91(4):919–30. 10.1099/vir.0.016766-0 PMC2888162 19955560

[68] Yao H, Lu X, Chen Q, et al. Patient-derived SARS-CoV-2 mutations impact viral replication dynamics and infectivity in vitro and with clinical implications in vivo. Cell Discov. 2020;6(1):76. 33298872 10.1038/s41421-020-00226-1 PMC7595057

[69] Velazquez-Salinas L, Zarate S, Eberl S, et al. Positive selection of ORF1ab, ORF3a, and ORF8 genes drives the early evolutionary trends of SARS-CoV-2 during the 2020 COVID-19 pandemic. Front Microbiol. 2020;11:550674. 10.3389/fmicb.2020.550674 PMC7644918 33193132

[70] Su YCF, Anderson DE, Young BE, et al. Discovery and genomic characterization of a 382-nucleotide deletion in ORF7b and ORF8 during the early evolution of SARS-CoV-2. mBio. 2020;11(4):e01610–20. 32694143 10.1128/mBio.01610-20 PMC7374062

[71] Gong YN, Tsao KC, Hsiao MJ, et al. SARS-CoV-2 genomic surveillance in Taiwan revealed novel ORF8-deletion mutant and clade possibly associated with infections in middle east. Emerg Microbes Infect. 2020;25(1):1754–1760. 10.1080/22221751.2020.1782271 PMC7473175 32543353

[72] Wang X, Lam JY, Wong WM, et al. Accurate diagnosis of COVID-19 by a novel immunogenic secreted SARS-CoV-2 orf8 protein. mBio. 2020;11(5):e02431–20. 33082264 10.1128/mBio.02431-20 PMC7587431

[73] Kijak GH, Sanders-Buell E, Chenine AL, et al. Rare HIV-1 transmitted/founder lineages identified by deep viral sequencing contribute to rapid shifts in dominant quasispecies during acute and early infection. PLoS Pathog. 2017;13(7):e1006510. 28759651 10.1371/journal.ppat.1006510 PMC5552316

[74] Piantadosi A, Freije CA, Gosmann C, et al. Metagenomic sequencing of HIV-1 in the blood and female genital tract reveals little quasispecies diversity during acute infection. J Virol. 2019;93(2):93. 10.1128/JVI.00804-18 PMC6321908 30381486

[75] Subissi L, Posthuma CC, Collet A, et al. One severe acute respiratory syndrome coronavirus protein complex integrates processive RNA polymerase and exonuclease activities. Proc Natl Acad Sci U S A. 2014;111(37):E3900–9. 25197083 10.1073/pnas.1323705111 PMC4169972

