# Supplementary material for: Quasispecies of SARS-CoV-2 revealed by single nucleotide polymorphisms (SNPs) analysis
Source: Virulence. 2021 May 25;12(1):1209–26. doi: 10.1080/21505594.2021.1911477 (PMC8158041; doi:10.1080/21505594.2021.1911477)
Supplement: Supplemental Material [file KVIR_A_1911477_SM2446.zip › legends.rtf]

Supplemental Information

Quasispecies of SARS-CoV-2 revealed by intra-host single nucleotide variations (iSNVs) analysis 
 
Rongsui Gao1,*, Wenhong Zu1,*, Yang Liu1,*, Junhua Li4,*, Zeyao Li6,*, Yanling Wen1, Haiyan Wang1, Yuxia Gao8, Jing Yuan2, Lin Cheng 1, Shengyuan Zhang1, Yu Zhang5, Shuye Zhang9, Weilong Liu1, Xun Lan7#, Lei Liu1#, Feng Li3#, Zheng Zhang1#


Supplementary figures

Figure S1. Allele composition and frequencies of SARS-CoV-2 SNPs.
A. The ratio of types of SNPs in this study and SNPs online(GISAID).
B. The ratio of types of C*>T* SNPs in our dataset and GISAID SNPs.
C. C>T mutations are distributed along the SARS-CoV-2 genome.


Supplementary Tables

Table S1. Primers used in this study.
 
Table S2. Clinical information of patients and samples. 
Patients with a dynamic component of quasispecies in samples are in red.

Table S3. Summary of SNPs of SARS-CoV-2 detected from samples in this study.

Table S4. Summary of SNPs of SARS-CoV-2 detected from online databases.
The shared SNPs were analyzed based on the SARS-CoV-2 genome sequences available online as of April 11, 2020 (2019nCoVR, https://bigd.big.ac.cn/ncov)

Table S5. The result of S glycoprotein peptides binding to MHC class I allele.  
The MHC class I binding prediction was made with the IEDB analysis resource using a consensus method that employs a Stabilized Matrix, Artificial Neural Networks, and combinatorial library methods (http://tools.iedb.org/tepitool/).


Supplementary Methods

Plasmid
The codon-optimized gene encoding SARS-CoV-2 S protein (GenBank: QHD43416) with C-terminal 19-amino acid deletion was synthesized by Sino Biological Inc (Beijing, China) and cloned into the NotI and ApaI restriction sites of pVAX vector (pVAX-SARS-CoV-2-S-C19del, denoted as pVAX-S). A series of mutant S-expressing plasmid (denoted as pVAX-S S359N, pVAX-S F486A, pVAX-S N487A, pVAX-S S494A, pVAX-S T500A, pVAX-S V503A, pVAX-S-motif-1 S494A&G496A&T500A&N501A&V503A&Q506A, pVAX-S-motif-2 F486A& N487A) was constructed by site-directed mutagenesis, with pVAX-S plasmid as a template. The codon-optimized genes encoding SARS-CoV-2 ORF8 were cloned into the BamHI and XhoI restriction sites of pCMV-GST vector (pCMV-GST-ORF8).The expression plasmid for human ACE2 was obtained from GeneCopoeia (Guangzhou, China). pCMV-HLA-A2 was obtained from Sino Biological Inc. (Beijing, China). The primers used for plasmid construction were listed in Table S1. 

Cell lines
HEK293T cells were purchased from the American Type Culture Collection (ATCC, Manassas, VA, USA). Cells were maintained in Dulbecco's Modified Eagle Medium (DMEM; Hyclone, Waltham, MA, USA) supplemented with 10% fetal bovine serum (FBS; Gibco, Rockville, MD, USA), 100 mg/mL of streptomycin, and 100 unit/mL of penicillin at 37°C in 5% CO2. HEK293T cells transfected with human ACE2 (293TACE2) were cultured under the same conditions with the addition of G418 (0.5 mg/mL) to the medium.
