# Supplementary figures and images for: Quasispecies of SARS-CoV-2 revealed by single nucleotide polymorphisms (SNPs) analysis
Source: Virulence. 2021 May 25;12(1):1209–26. doi: 10.1080/21505594.2021.1911477 (PMC8158041; doi:10.1080/21505594.2021.1911477)

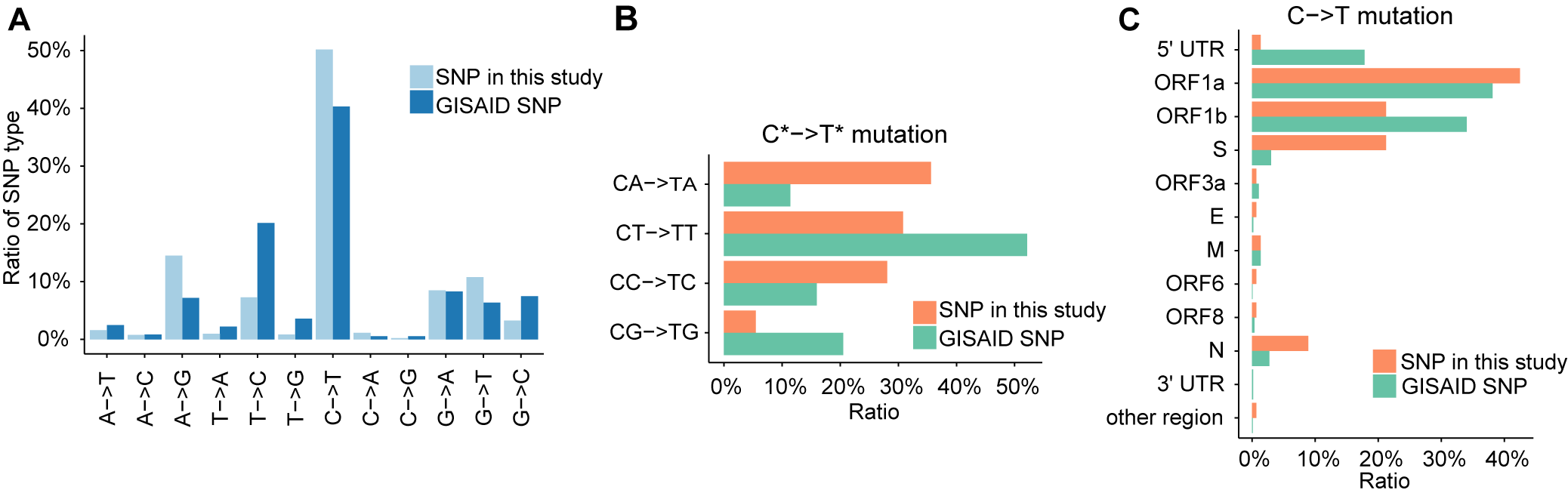

Supplement: Supplemental Material [file KVIR_A_1911477_SM2446.zip › Fig. S1.tif]
